# Social health organizations and their role in the diffusion of minimally invasive surgery in Brazil

**DOI:** 10.1016/j.clinsp.2026.101102

**Published:** 2026-09-06

**Authors:** Francisco Tustumi, Felipe Mendes Delpino, João Vitor Pincelli, Lucas Correa, Nelson Wolosker, Michael Anderson, Meskerem Kebede, Laia Maynou, Rachel Hargest, Rocco Friebel

**Affiliations:** a Department of Health Sciences, Hospital Israelita Albert Einstein, São Paulo, SP, Brazil; b Global Surgery Policy Unit, LSE Health, London School of Economics and Political Science, London, UK; c Universitat de Barcelona, Department of Econometrics, Statistics and Applied Economics (Public Policies), Barcelona, Spain

**Keywords:** Health policy, Public health, Public-private sector partnerships, Technology transfer, Diffusion of innovation

## Abstract

**Background:**

In Brazil, Social Health Organizations (SHOs), a form of public-private partnership, manage a growing number of public hospitals. This study assessed their role in the diffusion of laparoscopic cholecystectomy.

**Methods:**

In a nationwide study, the rates of laparoscopy were compared over time between SHO and non-SHO hospitals. We applied fixed-effects, Cox, and logistic regression models to examine the association between SHO and laparoscopy use, as well as the speed of laparoscopy adoption.

**Results:**

Of the 2208 hospitals assessed, 83 were SHO-managed. These hospitals exhibited higher laparoscopy rates for both elective and urgent procedures. SHO hospitals had a higher likelihood of reaching ≥ 30% laparoscopy adoption earlier (HR = 2.03; 95% CI: 1.84–2.23), and they reached the adoption threshold approximately one year earlier than non-SHO hospitals (mean time to adoption: 3.7 ± 5 vs. 4.5 ± 4.7 years, respectively).

**Conclusion:**

SHO-managed hospitals were associated with faster and broader adoption of laparoscopic surgery within the Brazilian public healthcare system. These findings support the potential of hybrid governance models to enhance the delivery of care in low- and middle-income settings, while acknowledging that residual confounding and selection bias cannot be fully excluded.

## Highlights

• Laparoscopic adoption for cholecystectomy remains uneven in Brazil’s public system.
• SHO-managed hospitals show faster growth in laparoscopic cholecystectomy use.
• SHOs reach key laparoscopy adoption thresholds earlier than non-SHO hospitals.
• SHOs adopt laparoscopic cholecystectomy earlier and more broadly.
• Public-private governance may facilitate minimally invasive surgery diffusion.

## Introduction

The diffusion of new medical technologies has substantially improved health outcomes.^1^ Among these innovations, laparoscopy stands out as a minimally invasive surgical technique that has become the gold standard for several gastrointestinal procedures, such as cholecystectomy and appendectomy.^2^ Compared to open surgery, laparoscopy is associated with faster recovery, reduced postoperative pain, shorter hospital stays, and quicker return to daily activities and work.^3^^,^^4^ Despite its well-established benefits, the use of laparoscopy remains uneven, especially in Low- and Middle-Income Countries (LMICs).^5^ Barriers, such as inadequate infrastructure, limited funding, and insufficient professional training, slow down its widespread adoption in resource-constrained healthcare systems.^6^

Among the main indications for abdominal surgery, cholecystitis is one of the most frequent reasons for emergency procedures^7^ In the United States, approximately 1 million cholecystectomies are performed annually, with around 90% of these procedures conducted laparoscopically.^8^^,^^9^ In Brazil, the majority of cholecystectomies are carried out within the public healthcare system, where open surgery remains the predominant approach. Between 2010 and 2022, a total of 2057,184 cholecystectomies were performed, of which 66% were open (laparotomic) procedures, and 34% were laparoscopic.^10^ In recent years, laparoscopic cholecystectomy was performed in 45% of cases in the Southeast (Brazil’s most developed region), compared to only 19% in the Northeast. This stark regional disparity highlights how the adoption of laparoscopy serves as an indicator for broader technological, financial, and technical capacities across Brazil.^10^^,^^11^

The Brazilian Unified Health System (or *Sistema Único de Saúde*; SUS) was established by the Federal Constitution and is characterized by universal coverage, public funding, and government-managed healthcare delivery.^12^ However, structural and operational limitations have led to performance disparities across the country. SUS receives only 44% of total health expenditures in Brazil, while the private sector accounts for 56%. Whereas countries with similar universal healthcare systems invest over US$ 3000 per capita annually, SUS spends approximately US$ 500 per capita annually. These disparities are compounded by regional inequalities. The North and Northeast regions have 40% fewer ICU beds than the other regions of the country.^13^ The Southeast region has a physician density of 3.7 per 1000 population, while the Northeast has only 1.7 physicians per 1000 inhabitants.^14^

To address these challenges, the government has increasingly partnered with Social Health Organizations (SHOs), nonprofit entities that manage public health facilities under contractual agreements. These organizations are responsible for staffing, maintenance, and service delivery in exchange for fixed monthly transfers and performance-based accountability metrics.^15^ In practice, SHOs operate under a management contract with the public sector, which clearly defines performance indicators, targets, and deadlines tailored to the specific health unit and public policy objectives. These contracts require the SHOs to provide regular reports on quality indicators, such as the number of patient consultations, volume of procedures, quality of services, and user satisfaction. These reports are closely monitored through audits and inspections. If the SHOs fail to comply with the agreed targets, they can receive corrective measures, ranging from action plans to financial penalties or even contract termination. This system ensures that SHO management is transparent, accountable, and aligned with the goals of the public health system.^16^

Theoretically, by coupling budgetary flexibility with performance-linked incentives, SHOs can redirect savings toward capital items that could improve surgical outcomes, such as laparoscopic towers for cholecystectomy, and fund rapid, on-site training that would otherwise be delayed by public-sector procurement rules. This SHO governance efficiency advantage could translate into faster adoption of minimally invasive surgery. As public-private partnership models similar to SHOs are increasingly adopted in various countries, evaluating their effectiveness in modernizing care delivery has become essential to guide evidence-based health policy and resource allocation decisions.^17^ However, although these proposed advantages are frequently invoked by governments and administrative organizations to justify the adoption of public-private management models, whether they translate into improved access to surgical innovation remains insufficiently supported by empirical evidence.

Despite the theoretical rationale, empirical evidence remains scarce on whether SHOs' operational efficiency enhances the adoption of surgical technologies, such as laparoscopy. In this context, we aim to examine differences in the adoption of laparoscopic cholecystectomy between SHO hospitals and non-SHO hospitals within Brazil’s public health system to ascertain if SHO hospitals are associated with higher rates of adoption of novel technology. Given laparoscopy's proven clinical and economic advantages, it offers a proxy for broader surgical modernization. Understanding whether and how public-private partnerships such as SHOs accelerate the diffusion of high-value surgical innovations could reshape health governance and funding priorities, especially in LMICs.

## Methods

We conducted a nationwide retrospective cohort analysis using data from three primary sources: the Brazilian Hospital Information System (SIH/SUS) provided by DATASUS, demographic population estimates from the Brazilian Institute of Geography and Statistics (i.e., *Instituto Brasileiro de Geografia e Estatística;* IBGE), and institutional information on SHOs from the Brazilian Institute of Social Health Organizations (i.e., *Instituto Brasileiro das Organizações Sociais de Saúde*; Ibross; accessed in April 2025). The study was reported in accordance with the STROBE (Strengthening the Reporting of Observational Studies in Epidemiology) guidelines.^18^

### Data sources and integration

We extracted microdata from SIH/SUS spanning from January 2011 to December 2024. This dataset includes all hospitalizations financed by SUS and contains information on patient demographics (e.g., gender, ethnicity, age), hospital characteristics, procedures performed, and dates of admission and discharge. We selected records corresponding to cholecystectomy procedures using the SIGTAP codes 0407,030,034 (laparoscopic) and 0407,030,026 (open). For each hospitalization, we derived the age from the date of birth, gender, ethnicity, elective versus non-elective status, and admission date, and categorized cases as elective or urgent. Hospitals were characterized by their annual procedure volume, the proportion of laparoscopic access, and the type of management (SHO or non-SHO). Population denominators for calculating procedure rates per 1000 inhabitants were obtained from IBGE population projections for each year. Specifically, we used the IBGE annual population estimates for each calendar year (2011–2024) to update denominators year by year, enabling longitudinal comparisons that account for demographic changes over time.

Hospitals managed by SHO were defined by cross-referencing the CNES (i.e., National Register of Health Establishments) codes from the SIH/SUS data with SHO management information obtained from the Ibross dataset. Records were excluded if they lacked valid hospital identifiers or SHO information.

Hospitals were classified as SHOs based on the official year of management contract initiation. SHO status was treated as a time-varying binary variable, allowing a given hospital to contribute observations both as non-SHO (prior to contract initiation) and as SHO (from the year of contract onward). This dynamic classification enabled the evaluation of changes within hospitals over time. By combining this variable with hospital fixed effects, the analysis controlled for all time-invariant hospital characteristics, isolating the association between SHO management and outcomes of interest.

We aggregated data at the hospital-year level according to age, gender, race, and proportion of urgent admissions. Race variables are compositional by definition, highly correlated, and prone to multicollinearity when entered simultaneously into regression models, particularly within a fixed-effects framework. To address these challenges and ensure robust adjustment for potential confounding by racial composition, we applied Principal Component Analysis (PCA) to the proportions of patients who self-identified as White, Black, Brown, Asian, or Indigenous. PCA is a data-reduction technique that transforms correlated variables into a smaller set of uncorrelated components that capture the dominant patterns of variation in the data. PCA was used to derive a single continuous index (PC1) summarizing the main gradient of racial distribution across hospitals, allowing parsimonious adjustment for demographic composition without imposing an arbitrary reference category. In our analysis, PC1 primarily contrasted higher proportions of White patients (loading: +0.69) with higher proportions of Brown patients (loading: −0.71), while other racial categories contributed minimally to this axis. This PCA-derived index was intended to capture broader structural and demographic gradients rather than subgroup-specific or causal effects of race.

Similarly, we applied PCA to the proportions of patients stratified into eight age-gender bands: female 18–39 years, female 40–64 years, female 65–74 years, female ≥75 years, male 18–39 years, male 40–64 years, male 65–74 years, and male ≥75 years. The first principal component captured a gradient across hospitals, contrasting those treating a higher proportion of younger female patients (strong negative loading for female 18–39: −0.77) with those treating a relatively higher proportion of middle-aged and older female and male patients (positive loadings: +0.56 for female 40–64, +0.21 for female 65–74, and smaller positive loadings for older male bands). This component was included as a continuous covariate in regression models to adjust for differences in hospital case mix, accounting for age and gender composition. As with race, this approach prioritized statistical stability and control of multicollinearity over intuitive subgroup-level interpretation, consistent with the study’s primary focus on governance effects rather than on estimating demographic effects.

We systematically evaluated missingness across all key variables. No imputation methods were applied because some variables ‒ particularly self-reported race ‒ were inconsistently recorded across hospitals and calendar years, suggesting a non-random pattern of missingness. Because race was included solely as an adjustment variable rather than a primary exposure, and because the missingness mechanism could not be reliably characterized, multiple imputation could have introduced additional bias or unwarranted precision. Admissions lacking hospital identifiers or procedure type (open vs. laparoscopic) were excluded. Self-reported race was included through the PCA approach, which accommodates partial missingness without requiring the imputation of individual categories. This strategy prioritized robustness of the primary estimates related to SHO management over potentially unstable recovery of demographic data. Variables with substantial missingness (e.g., secondary diagnoses and comorbidity codes) were excluded from multivariable models due to concerns regarding data completeness and interpretability. The high rate of missing entries made it unclear whether comorbidities were truly absent or underreported. To approximate differences in hospital case mix capacity and complexity, surgical volume was included as a proxy measure.

### Statistical analysis

We described the temporal evolution of laparoscopic access by SHO status and urgency. Laparoscopy rates were expressed as the percentage of laparoscopic cholecystectomies among all cholecystectomies and as rates per 1000 inhabitants. Population denominators were updated annually using IBGE estimates to incorporate year-to-year population changes. Baseline characteristics of patients and hospitalizations were compared between SHO and non-SHO hospitals. All comparisons were performed using the chi-squared test.

### Fixed effects regression

To evaluate the association between SHO status and laparoscopic access, we applied a fixed-effects linear regression model at the hospital-year level. The dependent variable was the annual rate of laparoscopic cholecystectomy per 1000 inhabitants. The model included SHO status (binary), year, an interaction term between SHO and year, and the following covariates: log-transformed surgical volume, racial composition, and age-gender composition. The proportion of urgent cases was also included. Fixed effects for hospital and year were included to control for unobserved, time-invariant hospital characteristics and time trends.

### Time to adoption

Hospitals were followed from their first year of recorded cholecystectomies until they reached a predefined adoption threshold (≥ 30% laparoscopic access) or were censored at the end of the observation period (December 2024). Adoption thresholds were defined as pragmatic markers of early and advanced diffusion stages rather than clinically validated targets, given the persistent use of open cholecystectomy in the Brazilian public health system and the limited number of hospitals that would meet higher thresholds during the study period.

Before fitting the Cox proportional hazards model, we evaluated the appropriateness of time-to-event modeling by confirming adequate variation in follow-up time and enough adoption events to ensure stable hazard estimation. No structural features were identified that would contraindicate survival modeling. The proportional hazards assumption was formally tested using Schoenfeld residuals. For SHO status and the overall model, the tests yielded p-values > 0.05, indicating no statistical evidence of violation of the proportional hazard’s assumption.

A Cox proportional hazards model was applied to estimate the association between SHO status and time to adoption. The model adjusted for potential differences in hospital case-mix, including log-transformed surgical volume, the proportion of urgent procedures, a principal component representing racial composition, and a principal component summarizing age-gender composition. The survival object incorporated time (in years) and event status (reached ≥ 30%). Kaplan-Meier curves were used to compare cumulative adoption rates between SHO and non-SHO hospitals. To ensure the robustness of the findings, we conducted a sensitivity analysis using a higher threshold of laparoscopic adoption (≥ 50%).

### Early vs. late adopters

Hospitals with <2-years of follow-up were excluded from this analysis. Among the remaining, those achieving ≥ 30% laparoscopic access within 2-years were classified as Early Adopters, while others were labeled Late Adopters. A logistic regression model was used to identify predictors of early adoption, adjusting for type of hospital management (SHO vs. non-SHO), log-transformed surgical volume, the proportion of urgent procedures, a principal component summarizing racial composition, and a principal component summarizing age-gender composition across eight predefined age-gender bands. As a sensitivity analysis, the same approach was repeated using a higher adoption threshold (≥50%).

All analyses were conducted using RStudio (version 4.2.0). A two-sided significance level of p < 0.05 was considered statistically significant.

## Results

### Baseline characteristics of the patients

The study included 1549,851 hospital admissions for cholecystectomy between 2011 and 2024, with a mean patient age of 46.1-years (±15.6). Among the procedures performed, 952,739 were open (61.5%), and 597,112 (38.5%) were laparoscopic. Most admissions were elective (1125,766; 72.6%), while 424,064 (27.4%) were urgent. Regarding self-reported race, 45.6% of patients were classified as Brown (Mixed), 26.9% White, 3.5% Black, 2.0% Asian, and 0.2% Indigenous. Race data were missing for 21.8% of cases.

A total of 2208 hospitals were included in the analysis, of which 83 (3.7%) were managed by SHOs. The mean SHO experience, defined as the number of years since the beginning of the management contract, was 10.6-years (±5.6). The baseline characteristics of the patients by hospital management type (SHO vs. non-SHO) are summarized in Table 1.

**Table 1:** Baseline characteristics of the included patients admitted to hospitals managed by Social Health Organizations (SHO) or traditional public administration.

Characteristic	Non-SHO (2125 hospitals)	SHO (83 hospitals)	p-value
**Patient admissions (N)**	1433,885	115,966	
**Surgical Access**			<0.001
Open	902,133 (63%)	50,606 (44%)	
Laparoscopic	531,752 (37%)	65,360 (56%)	
**Admission Type**			<0.001
Elective	1051,836 (73%)	73,930 (64%)	
Urgent	382,028 (27%)	42,036 (36%)	
Unknown	21	0	
**Age group**			<0.001
18‒39	503,021 (36%)	39,484 (35%)	
40‒64	714,381 (51%)	57,101 (50%)	
65‒74	141,835 (10%)	12,551 (11%)	
75+	48,990 (3.5%)	4931 (4.3%)	
Unknown	25,658	1899	
**Gender**			<0.001
Female	1156,268 (81%)	92,300 (80%)	
Male	277,617 (19%)	23,666 (20%)	
**Race**			<0.001
Black	48,042 (4.3%)	5516 (6.3%)	
Brown	646,780 (57%)	60,049 (69%)	
Indigenous	3549 (0.3%)	57 (<0.1%)	
White	396,460 (35%)	20,152 (23%)	
Asian	30,166 (2.7%)	1375 (1.6%)	
Unknown	308,888	28,817	

Column headers indicate the number of hospitals in each group, while table rows report patient-level counts and percentages. Values are presented as counts and percentages. p-values were calculated using Pearson’s Chi-Squared test. Age was calculated as the integer number of years based on the date of birth and admission date. Surgical access was classified as open or laparoscopic (the DATASUS database does not provide information regarding conversion). Unknown values were excluded from comparative tests. Red p-values indicate significant associations (p < 0.05).

### Use of laparoscopy over time

**Fig. 1:**
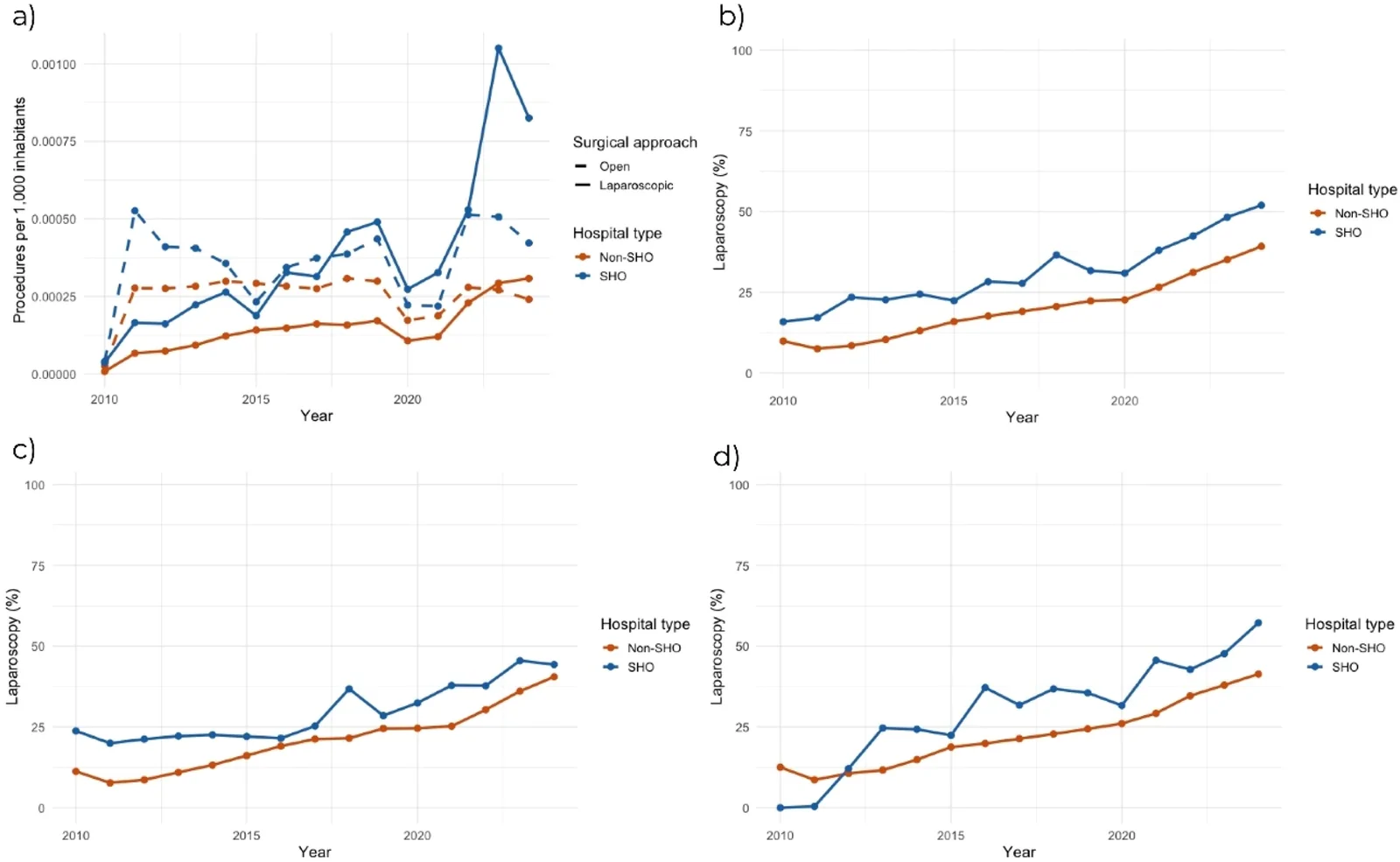
Use of laparoscopy for cholecystectomy over time. (a) Number of cholecystectomies per 1000 inhabitants by hospital type and surgical approach; (b) Annual average use of laparoscopy by type of hospital management; (c) Use of laparoscopy in urgent cholecystectomies by hospital type; (d) Use of laparoscopy in elective cholecystectomies by hospital type. SHO, Social Health Organization.

The use of laparoscopic cholecystectomy increased steadily over the study period across the Brazilian public healthcare system. Fig. 1a shows population-based rates of cholecystectomy per 1000 inhabitants, stratified by hospital management type and surgical approach, showing a progressive increase in laparoscopic procedures over time, alongside relative stability in open surgery. Panels 1b–1d present hospital-level proportions of laparoscopic use, illustrating differences in practice patterns by hospital management type. SHO-managed hospitals had higher average proportions of laparoscopic cholecystectomy over time than non-SHO hospitals (Fig. 1b). Similar patterns were observed when procedures were stratified by urgency, with greater laparoscopic use in both urgent (Fig. 1c) and elective (Fig. 1d) cholecystectomies among SHO hospitals.

### Fixed-effects analysis

The fixed-effects linear regression model revealed that hospitals managed by SHOs were significantly associated with higher rates of laparoscopic cholecystectomy over time. Specifically, while the SHO variable alone showed a negative association with the outcome (p = 0.0017), its interaction with the year was positively associated with the laparoscopic rate (p = 0.0017), indicating a steeper increase in adoption among SHO-managed hospitals. Hospital surgical volume was also positively associated with laparoscopic cholecystectomy rates (p < 0.001). Other covariates, such as the proportion of urgent admissions, the first principal component of racial composition, and the first principal component of age-gender bands, did not show significant associations (see Table 2).

**Table 2:** Coefficients from fixed-effects linear regression model with hospital-year laparoscopic cholecystectomy rate per 1000 inhabitants as the dependent variable. The model includes hospital and year fixed effects, with standard errors clustered at the hospital level. Case-mix adjustment was performed using principal components of race and age-gender bands and hospital surgical volume. A positive coefficient indicates an association with higher laparoscopy use. Within R² = 0.0674. Red p-values indicate significant associations (p < 0.05).

Variable	Estimate	95% CI lower	95% CI upper	p-value
SHO hospital	−0.0714	−0.116,064	−0.026,731	0.0017
Log surgical volume	0.000,115	0.000,097	0.000,133	<0.001
Proportion of urgent admissions	−0.00,001	−0.000,044	0.000,024	0.5664
Race (PCA)	−0.000,005	−0.000,017	0.000,008	0.4669
Age-gender bands (PCA)	−0.000,002	−0.000,006	0.000,001	0.2236
SHO × Year interaction	0.000,035	0.000,013	0.000,057	0.0017

### Time-to-adoption

**Fig. 2:**
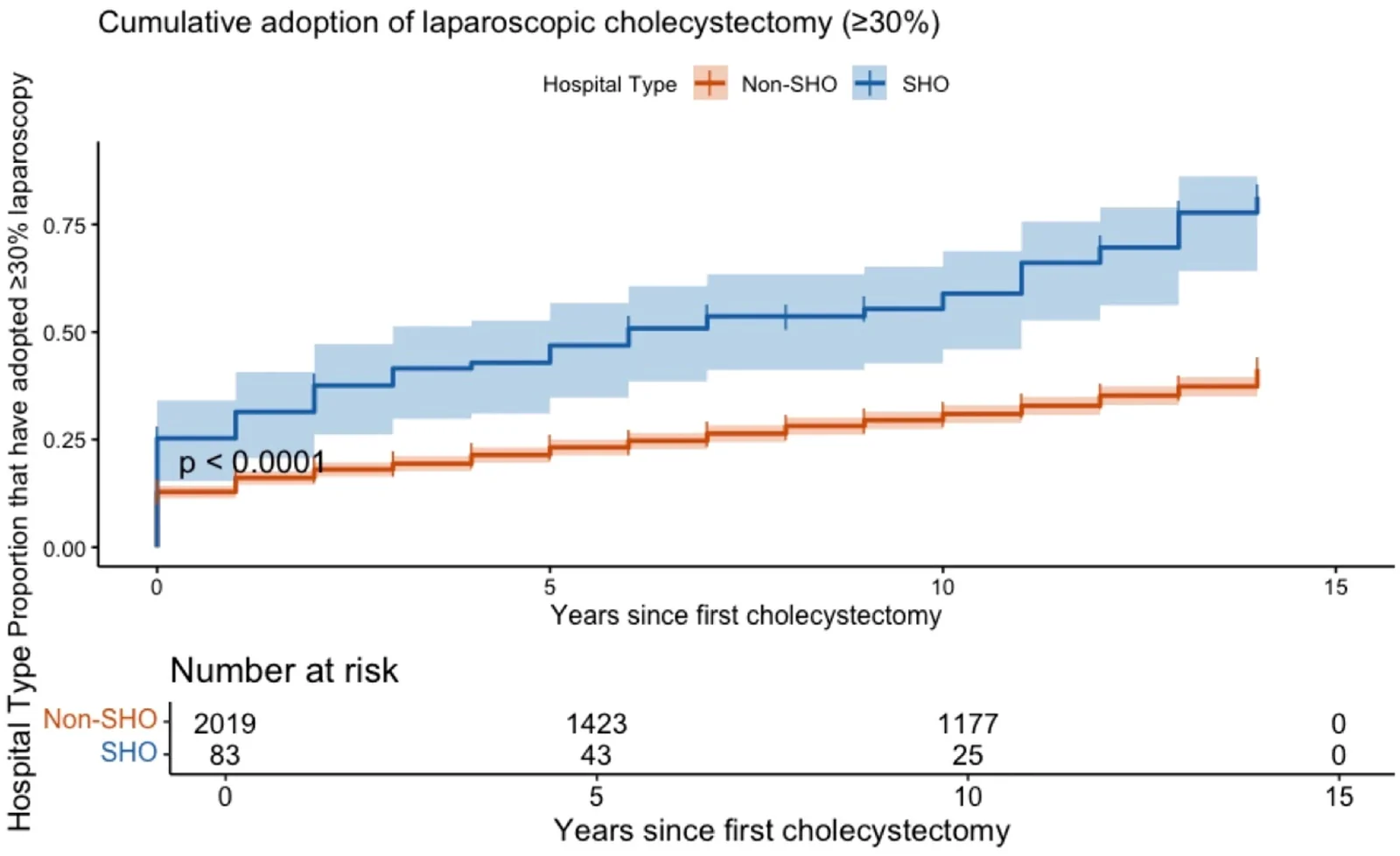
Kaplan-Meier cumulative incidence curve comparing the time to adoption of laparoscopic cholecystectomy (≥ 30%) between hospitals managed by Social Health Organizations (SHO) and those under traditional public administration (Non-SHO).

**Fig. 3:**
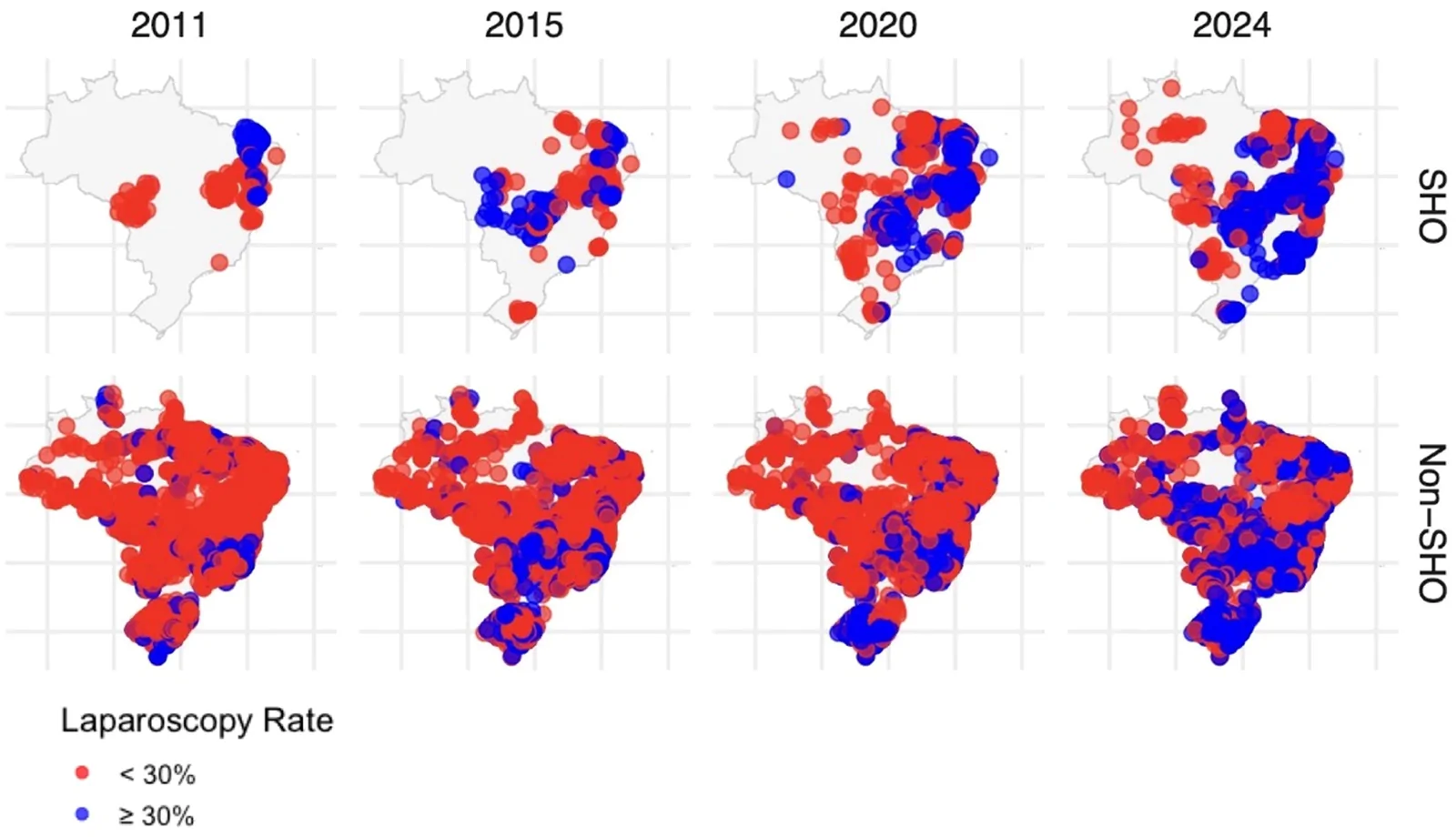
Geographic distribution of hospitals by laparoscopic cholecystectomy adoption rate (≥ 30%) in Brazil across selected years (2011, 2015, 2020, and 2024). Each point represents a hospital performing cholecystectomies, color-coded by adoption status: blue for hospitals with ≥ 30% laparoscopic use and red for those with < 30%. Maps are stratified by hospital management type: Social Health Organizations (SHO) vs. Non-SHO. The maps illustrate both temporal progression and regional disparities in the diffusion of laparoscopic surgery.

In the multivariable Cox regression analysis, hospitals managed by SHOs demonstrated a significantly faster adoption of laparoscopic cholecystectomy at or above the 30% threshold compared to non-SHO institutions (HR=2.03; 95% CI 1.84–2.23; p < 0.001; see Fig. 2, Fig. 3). Higher surgical volume was also independently associated with earlier adoption (HR = 1.52; 95% CI 1.49–1.55; p < 0.001). A higher proportion of urgent procedures was positively associated with earlier adoption (HR = 1.40; 95% CI 1.28–1.53; p < 0.001). In addition, racial composition was a statistically significant predictor of earlier adoption (HR = 1.17; 95% CI 1.15–1.19; p < 0.001). The PCA loadings indicated that hospitals treating a higher proportion of White patients were more likely to adopt laparoscopy sooner, while those serving a larger proportion of Brown patients were associated with slower adoption. Similarly, the age-gender composition was associated with faster adoption (HR = 1.86; 95% CI 1.76–1.96; p < 0.001). Hospitals with a higher proportion of middle-aged and older female patients (ages 40–64 and 65–74) were more likely to adopt laparoscopic techniques earlier. The model's concordance statistic was 0.711. The results of the sensitivity analysis using a 50% laparoscopic adoption threshold were consistent with those observed for the 30% threshold (the model's concordance statistic was 0.705). See Table 3.

**Table 3:** Cox proportional hazards models evaluating predictors of time to adoption of laparoscopic cholecystectomy at ≥ 30% and ≥ 50% thresholds, and logistic regression estimating the odds of early adoption of laparoscopic cholecystectomy, defined as achieving the respective threshold (≥ 30% or ≥ 50%) within < 2-years.

Adoption of laparoscopy								
	≥ 30%				≥ 50%			
		95% CI				95% CI		
**Variable**	**HR**	**Lower**	**Upper**	**p-value**	**HR**	**Lower**	**Upper**	**p-value**
SHO hospital	2.03	1.84	2.23	<0.001	2.10	1.91	2.32	<0.001
Log surgical volume	1.52	1.49	1.55	<0.001	1.47	1.44	1.50	<0.001
Proportion of urgent procedures	1.40	1.28	1.53	<0·001	1.27	1.16	1.40	<0.001
Race (PCA)	1.17	1.15	1.19	<0.001	1.18	1.16	1.21	<0.001
Age-gender bands (PCA)	1.86	1.76	1.96	<0.001	1.86	1.77	1.97	<0.001
**Early adoption of laparoscopy**								
	**≥ 30%**				**≥ 50%**			
		**95% CI**				**95% CI**		
**Variable**	**OR**	**Lower**	**Upper**	**p-value**	**OR**	**Lower**	**Upper**	**p-value**
SHO hospital	3.67	1.92	6.89	<0.001	3.33	2.57	4.30	<0.001
Log surgical volume	1.39	1.29	1.51	<0.001	1.53	1.47	1.59	<0.001
Proportion of urgent cases	0.72	0.47	1.11	0.144	1.49	1.24	1.79	<0.001
Race (PCA)	1.12	1.03	1.22	0.011	1.18	1.13	1.23	<0.001
Age-gender bands (PCA)	1.79	1.48	2.18	<0.001	2.10	1.88	2.34	<0.001

HR, Hazard Ratio; ORs, Odds Ratios; CI, Confidence Interval; SHO, Social Health Organization; PCA, Principal Component Analysis. Significant p-values (p < 0·05) are highlighted in red.

Among hospitals that reached the 30% adoption threshold, SHO hospitals adopted laparoscopic cholecystectomy more rapidly, with a mean time to adoption of 3.7 ± 5.0 years, compared to 4.5 ± 4.7 years among non-SHO hospitals. Similarly, for the 50% threshold, the mean time to adoption was 4.1 ± 5.1 years for SHO hospitals and 4.6 ± 4.8 years for non-SHO hospitals.

### Early vs. late adopters

Among all hospitals, 15% were classified as early adopters at the ≥30% threshold, having reached a laparoscopic adoption rate of at least 30% within the first two years of their cholecystectomy series, while 85% were classified as late adopters. In the multivariable logistic regression, SHO hospitals had significantly higher odds of being early adopters (OR = 3.67; 95% CI 1.92–6.89; p < 0.001). Greater surgical volume (log-transformed) was positively associated with early adoption (OR = 1.39; 95% CI 1.29–1.51; p < 0.001). The racial composition principal component was independently associated with earlier adoption (OR = 1.12; 95% CI 1.03–1.22; p = 0.011), as was the age-gender composition (OR = 1.79; 95% CI 1.48–2.18; p < 0.001). The proportion of urgent cases was not significantly associated with early adoption at this threshold (OR = 0.72; 95% CI 0.47–1.11; p = 0.144).

In the sensitivity analysis using a ≥ 50% threshold, 12.6% of hospitals were classified as early adopters. In this model, SHO hospitals again showed higher odds of early adoption (OR = 3.33; 95% CI 2.57–4.30; p < 0.001), as did greater surgical volume (OR = 1.53; 95% CI 1.47–1.59; p < 0.001). Notably, the proportion of urgent procedures was significantly associated with early adoption at this higher threshold (OR = 1.49; 95% CI 1.24–1.79; p < 0.001), suggesting that hospitals achieving high levels of laparoscopic penetration were those capable of expanding laparoscopy to urgent settings. The racial composition (OR = 1.18; 95% CI 1.13–1.23; p < 0.001) and age-gender composition (OR = 2.10; 95% CI 1.88–2.34; p < 0.001) remained strong independent predictors of early adoption at the ≥ 50% threshold.

## Discussion

This study provides evidence on the diffusion of laparoscopic cholecystectomy in the Brazilian public healthcare system, with a particular focus on the role of SHOs. By analyzing nationwide data from 2011 to 2024, we observed that SHO-managed hospitals consistently demonstrated higher rates of laparoscopic adoption compared to non-SHO institutions. Although these findings should be interpreted strictly within an observational framework, given the non-random assignment of hospitals to SHO management and the possibility of residual confounding, rather than as evidence of a causal effect, and should be understood as average associations across heterogeneous institutional arrangements, this study brings relevant empirical insights into patterns of surgical technology diffusion in a large middle-income country.

This study deliberately combined population-based rates and hospital-level proportions to capture complementary dimensions of laparoscopic diffusion. Population-based rates, calculated per 1000 inhabitants using annual IBGE population estimates, were used in the main longitudinal analyses to reflect system-level access to laparoscopic cholecystectomy over time, incorporating demographic variation, service capacity, and population demand. In contrast, hospital-level proportions were used descriptively to illustrate intra-hospital practice patterns independent of population dynamics. While proportions more directly reflect institutional decision-making, they are sensitive to volume fluctuations and do not capture whether access to laparoscopic surgery is expanding or contracting at the population level. Accordingly, population-based rates were prioritized for policy-oriented analyses, whereas proportions were presented for descriptive visualization.

In multivariable fixed-effects regression, SHO status was independently associated with a higher annual growth rate in laparoscopic use. Similarly, in Cox regression, SHO hospitals were more likely to reach the 30% adoption threshold earlier. On average, SHO hospitals reached the 30% adoption threshold approximately one year earlier than non-SHO hospitals (mean time to adoption: 3.7 ± 5.0 years vs. 4.5 ± 4.7 years, respectively). These associations remained significant even after adjusting for hospital volume, demographic factors, urgency case-mix, and a composite racial/ethnic index. Additionally, SHO hospitals were more likely to serve as early adopters, reinforcing their leading role in the diffusion of surgical innovation. However, because hospitals were not randomly assigned to SHO management, these associations may reflect, at least in part, pre-existing institutional advantages or divergent performance trajectories rather than the effect of governance reform alone. Moreover, SHO management was operationalized as a binary indicator and does not capture heterogeneity in contract scope, funding levels, managerial capacity, or performance incentives, which may vary substantially across institutions.

SHOs are a form of public-private partnership to enhance the efficiency and flexibility of hospital management within the Brazilian public health system. Unlike traditionally managed public hospitals, SHOs operate under performance contracts, which allow for greater autonomy in hiring, procurement, and technological investments.^19^ This flexibility may contribute to faster adoption of surgical innovations by facilitating the recruitment of skilled professionals, reducing bureaucratic delays, and fostering an environment that values outcome-driven practices. However, these proposed mechanisms remain hypothetical in the context of this study’s design and cannot be directly tested using the available administrative data. Our analysis does not include mediation models, qualitative data, or institutional process measures that would allow formal evaluation of these pathways. Consequently, while such mechanisms may help contextualize the observed associations, they do not establish causality. Identifying and validating causal pathways would require mixed methods approaches, including qualitative institutional analyses and prospective evaluations.

In light of these limitations in testing underlying mechanisms, we also explored whether alternative longitudinal analytical strategies could better isolate changes associated with the introduction of SHO management. However, most SHO hospitals either began operating already under SHO governance or transitioned at very early stages of hospital activity, resulting in insufficient pre- and post-transition observations. Under these conditions, a difference-in-differences analysis would be underpowered and potentially misleading and was therefore not pursued.

Several countries have adopted comparable models to Brazil’s SHOs by using Public-Private Partnership (PPP) strategies for healthcare management. In the United Kingdom, NHS Foundation Trusts are semi-autonomous units that remain publicly owned, allowing them to have flexible service contracts and reinvest surpluses.^20^ In the United States, many hospitals are publicly funded but managed by private non-profit entities. Denver Health, for example, relies on a hybrid type of funding. It receives funding from public sources, including Medicare, Medicaid, taxpayer dollars, and private donations through the Denver Health Foundation, and serves mostly vulnerable populations.^21^

Several governments in LMICs have also promoted partnerships to align private institutions with public health goals. However, the discussion about the role of PPP in healthcare persists worldwide. In Brazil, the SHO model itself has been the subject of sustained political and legal debate, with critics arguing that it may represent a form of indirect privatization of public services, while proponents emphasize gains in managerial flexibility, efficiency, and accountability under performance-based contracts. This tension reflects broader global debates about the appropriate balance between public oversight and private managerial autonomy in healthcare systems. In 2018, one of the main Spanish public-private administrative concessions, the Alzira model, was terminated, generating intense political debates.^22^ Taken together, these international experiences illustrate both the potential benefits and the persistent controversies associated with PPP-based governance models. Importantly, each PPP arrangement has distinct legal, contractual, and political contexts, which limit direct comparability and constrain the external validity and generalizability of findings from the Brazilian SHO model.

Nevertheless, PPP models have been increasingly implemented to enhance healthcare delivery, technology transfer, and innovation diffusion globally, particularly in resource-constrained settings. These alliances bring together private sector expertise in manufacturing, distribution, marketing, and business planning with public infrastructure.^23^ Product Development Partnerships (PDPs) are among the most impactful global PPPs. PDPs are non-profit research and development collaborations funded by public and philanthropic institutions aimed at developing and ensuring equitable access to health technologies for neglected diseases.^24^ By operating pharmaceutical and diagnostic development, PDPs bring together academia and the private sector and are pivotal in transferring technology to LMICs. PDPs have enabled over 60 new health technologies, reaching >2.4 billion people globally.

Interestingly, the proportion of urgent procedures showed a differential impact depending on the adoption threshold. In the early vs. late adoption analysis using the ≥30% threshold, this variable was not significantly associated with early adoption. However, at the ≥50% threshold, a higher proportion of urgent procedures became a strong predictor of early adoption (OR=1.49, <0.001). This pattern likely reflects the natural progression of laparoscopic diffusion within hospitals. In the initial stages of adoption, laparoscopic cholecystectomy is typically prioritized for elective cases, where operative conditions are more controlled. Urgent surgical care, particularly in resource-constrained environments, is often less standardized, with a high level of variability, and more reliant on the availability of on-call teams and surgical equipment. Facilities with a high burden of emergency cases may face structural limitations that hinder the consistent use of laparoscopy in acute settings.^25^^,^^26^ Furthermore, clinical hesitancy regarding laparoscopic surgery in urgent patients, especially in the early stages of technology dissemination, may have contributed to delayed adoption in these hospitals. As institutions mature in their laparoscopic capabilities and move toward higher adoption rates, they increasingly extend the technique to more urgent and complex cases. Therefore, the significant role of urgent procedures at the ≥50% threshold suggests that achieving higher levels of laparoscopic penetration depends on a hospital’s ability to integrate minimally invasive approaches into emergency surgery. Importantly, the adoption thresholds used in this study (≥30% and ≥50% laparoscopic cholecystectomy) should be interpreted as analytical benchmarks rather than clinically normative targets. Although a 30% laparoscopic rate would be considered low in many contemporary health systems, the Brazilian public context is characterized by substantial persistence of open cholecystectomy in public hospitals. Under these conditions, very high thresholds would yield few hospitals reaching the endpoint, resulting in unstable and underpowered analyses. The selected cutoffs were therefore intended to capture earlier and later stages of the diffusion curve.

A key finding of our study was the observed association between the age-gender composition of hospitals and the speed of laparoscopic adoption. The PCA-derived index summarizing hospital age-gender composition was a significant predictor of both earlier adoption and early adopter classification. Hospitals serving a higher proportion of older patients (particularly women aged 40-years and older and men across all age groups) were more frequently observed among hospitals adopting laparoscopy earlier, while those serving predominantly young female populations were slower in this process. Although the observational nature of our analysis does not allow causal interpretation, some epidemiological patterns of gallstone disease may offer plausible contextual explanations for this finding. Women are more likely to develop gallstones, often at younger ages (20–50 years), and many elective cholecystectomies in women occur in this younger cohort.^27^^,^^28^ In contrast, men tend to present at older ages, frequently with a higher risk of complications.^29^^,^^30^ Older age is also associated with higher surgical risk, and in this context, the benefits of minimally invasive approaches (including lower morbidity, faster recovery, and reduced hospital stay) are particularly compelling.^31^^,^^32^ Hospitals treating older and more complex patient populations may, therefore, face more substantial institutional incentives to adopt laparoscopic techniques to optimize outcomes. Conversely, institutions with a younger, predominantly female patient base may experience less clinical or operational pressure to rapidly shift toward laparoscopy, especially if mainly treating low-risk elective cases where open surgery was still acceptable in earlier years of the diffusion curve. However, these clinical considerations should be interpreted only as potential contextual factors rather than definitive explanations for the observed association. Because the PCA variable represents a composite demographic summary measure, the observed relationship may also reflect unmeasured institutional characteristics, differences in case-mix complexity, or other structural factors not captured in administrative datasets. In addition, collinearity between covariates in the multivariable models may have led to over-adjustment. For instance, hospitals with a higher proportion of male patients may also have higher rates of urgent procedures, a variable already included and adjusted for in the models, leaving a residual effect in the opposite direction for sex. Additionally, a key limitation of this study is the lack of data on laparoscopy conversions. Hospitals with a higher proportion of male patients could have a higher rate of conversion to open cholecystectomy since conversion is more common in men. Therefore, the association between age–gender composition and the speed of laparoscopic adoption should be interpreted cautiously and warrants further investigation in future studies.

Racial composition appeared to influence the adoption of laparoscopy, as reflected by the PCA-derived race component. In our models, a positive association with PCA indicates that hospitals with a higher proportion of White patients were more likely to adopt laparoscopy earlier or at higher rates, whereas hospitals serving more Brown populations lagged behind. This may reflect broader structural inequalities in the Brazilian healthcare system, where race intersects with geography and socioeconomic status. The racial composition of the patient population was also significantly associated with the adoption of laparoscopy. In our analysis, hospitals with higher PCA scores (indicating a greater proportion of White patients) were more likely to adopt laparoscopy earlier. This result highlights potential structural inequities in access to surgical innovations, possibly reflecting broader socioeconomic disparities correlated with racial demographics. Importantly, this PCA-based variable should be interpreted as a proxy for underlying structural and socioeconomic inequalities rather than as a biological or causal effect of race itself. This finding reinforces the importance of examining racial equity in the dissemination of surgical technologies in future studies.^33^

At the same time, from a policy perspective, these findings raise a critical equity dilemma: if SHO models are preferentially implemented in already advantaged hospitals or regions, they may accelerate the local diffusion of innovation while potentially widening inter-hospital and regional disparities in access to minimally invasive surgery. Our data do not allow us to determine whether SHOs mitigate or exacerbate these inequities, underscoring the need for equity-sensitive implementation strategies and for future evaluations explicitly designed to address this question. Consequently, caution is warranted when extrapolating these findings to broader political initiatives or to the unrestricted expansion of SHO or other PPP-based management models. In addition, policies aimed at accelerating laparoscopic diffusion through PPPs may entail opportunity costs, including the diversion of financial, human, or infrastructure resources from other essential services, as well as increased short-term expenditures for equipment acquisition and training. Future research should evaluate the comparative cost-effectiveness of SHO and non-SHO hospitals from the public payer perspective, assessing which governance model offers greater economic value for publicly funded healthcare systems.

Surgical volume was a key variable in our analysis and was consistently associated with earlier and higher adoption of laparoscopy. Higher-volume hospitals are more likely to concentrate surgical expertise, institutional infrastructure, and better resource allocation, which may facilitate the implementation of advanced techniques. To address the right-skewed distribution typically observed in procedural volume data, we applied a log transformation, which enabled more accurate modeling of its relationship with laparoscopic uptake and mitigated the influence of outliers. Nevertheless, our ability to assess the complexity of surgical cases was limited. Although the DATASUS database includes some fields related to comorbidities, there was significant missing data and a lack of granularity regarding disease severity. As a result, we could not fully capture the clinical case-mix beyond hospital volume, demographic characteristics, and urgency of hospitalization. This limitation underscores the need for more detailed administrative data to better isolate the effect of organizational factors, such as SHO management, from potential confounding due to patient complexity.

### Strengths and limitations

This retrospective longitudinal study evaluated a comprehensive national dataset spanning 2011 to 2024, encompassing over 1.5 million cholecystectomy admissions across 2208 public hospitals in Brazil. The large sample size, broad temporal coverage, and nationwide scope provided a unique opportunity to robustly assess both temporal trends and institutional determinants of laparoscopic adoption in a real-world, heterogeneous healthcare system. By capturing data from virtually the entire Brazilian public sector, this study offers one of the most extensive analyses on the diffusion of minimally invasive surgery within a middle-income country context.

Nevertheless, this study has some limitations. The primary limitation of this study is the non-random assignment of hospitals to SHO management, which raises concerns regarding selection bias and residual confounding. Hospitals selected for SHO management may differ systematically from non-SHO hospitals in ways that are not fully observable in administrative data, including pre-existing performance trajectories, institutional capacity, or concurrent managerial and political factors. Although we employed hospital fixed-effects models to control for time-invariant characteristics, unmeasured and time-varying confounders cannot be fully excluded. Consequently, the observational design precludes causal inference, and the findings should be interpreted as hypothesis-generating.

Additional limitations should be acknowledged. The analysis relied on population-based administrative data, which may be subject to inaccuracies in registration or missing variables not captured in the database, such as surgeon-level information, technical resources, including the number and functionality of laparoscopic towers, the timing of equipment acquisition, access to disposable instruments, surgeon-level operative volume, training or certification, and institutional performance or remuneration incentives that could influence the choice of surgical approach, or detailed measures of hospital complexity. Although several hospital-level characteristics were adjusted for, including surgical volume and case-mix proxies, these variables cannot fully account for differences in clinical complexity or resource intensity across institutions. In this context, the modest within-hospital explained variance (within R²) observed in the fixed-effects models should be interpreted as reflecting the complex and multifactorial nature of laparoscopic adoption. The decision to adopt laparoscopy is influenced by multiple time-varying factors not captured in administrative datasets ‒ such as surgeon expertise and training, availability of laparoscopic equipment, local leadership and organizational culture, and episodic investments in infrastructure ‒ that may change within the same hospital over time. Future studies incorporating surgeon-level variables, such as training background, laparoscopic expertise, and certification, as well as hospital-level information on laparoscopic infrastructure and performance-based incentives, may help better characterize these remaining sources of unmeasured confounding and further clarify institutional determinants of laparoscopic adoption. While fixed-effects models are designed to estimate within-hospital associations net of time-invariant characteristics, they are not intended to fully explain all sources of variation in clinical practice patterns.

In addition, our definition of SHO status was based on the year of contract initiation, which may not reflect nuances in contract implementation, renewal, or operational maturity over time. In addition, both the binary operationalization of SHO status and the use of adoption thresholds represent necessary simplifications imposed by the available data and should be interpreted accordingly. Finally, this study did not include a cost analysis. Although increased adoption of laparoscopy may reflect improved access to minimally invasive surgical care, it may also entail additional expenditures for equipment acquisition and maintenance, disposable instruments, staff training, and organizational implementation. The available administrative data do not allow assessment of whether the faster diffusion observed among SHO-managed hospitals was associated with greater costs or represented an economically efficient allocation of public resources. Future studies should incorporate comparative cost and cost-effectiveness analyses of SHO and non-SHO hospitals from the perspective of the public payer.

## Conclusion

SHO-managed hospitals were associated with faster diffusion of laparoscopic surgery within the Brazilian public healthcare system. SHO’s management structure may offer critical advantages that promote faster and broader adoption of surgical innovations. These findings support the potential of hybrid governance models to enhance the delivery of surgical care in low- and middle-income settings, while acknowledging that residual confounding and selection bias cannot be fully excluded.

## Ethical approval

Ethical approval for this type of study is not required by our institute.

## Institutional review board statement

Not applicable.

## Informed consent statement

Not applicable.

## Data availability

The corresponding author will provide the datasets used and/or analyzed during the current work upon reasonable request.

## Authors’ contributions

Francisco Tustumi: Conceptualization; Methodology; Formal analysis; Investigation; Writing-original draft, Writing-review & editing; Supervision; Project administration.

Felipe Mendes Delpino: Methodology; Formal analysis; Data curation; Writing-review & editing; Visualization.

João Vitor Pincelli: Investigation; Data curation, Writing-review & editing.

Lucas Correa: Methodology; Formal analysis; Writing-review & editing.

Nelson Wolosker: Conceptualization; Supervision; Writing-review & editing.

Michael Anderson: Methodology; Validation; Writing-review & editing.

Meskerem Kebede: Investigation; Validation; Writing-review & editing.

Laia Maynou: Formal analysis; Methodology; Writing-review & editing.

Rachel Hargest: Validation; Supervision; Writing-review & editing.

Rocco Friebel: Conceptualization; Methodology; Formal analysis; Supervision; Funding acquisition; Writing-review & editing.

## Funding

This study did not receive any specific funding.

## Declaration of competing interest

Dr. Francisco Tustumi and Felipe Mendes Delpino received research scholarships from the Bracell Foundation. The foundation had no role in the study design, data collection, analysis, interpretation, manuscript preparation, or the decision to submit this work for publication. All other authors declare no conflicts of interest, financial or otherwise. The research was conducted independently, without any sponsor involvement.

## Appendix Supplementary materials

Image, application 1
